# Evaluation of a Single Dose of Azithromycin for Trachoma in Low-Prevalence Communities

**DOI:** 10.1080/09286586.2017.1293693

**Published:** 2018-12-13

**Authors:** Nana Wilson, Brook Goodhew, Harran Mkocha, Kahaliah Joseph, Claudiu Bandea, Carolyn Black, Joseph Igietseme, Beatriz Munoz, Sheila K. West, Patrick Lammie, Mabula Kasubi, Diana L. Martin

**Affiliations:** a Division of Parasitic Diseases and Malaria, Centers for Disease Control and Prevention, Atlanta, GA, USA; b Kongwa Trachoma Project, Kongwa, Tanzania; c National Center for Emerging, Zoonotic, and Infectious Diseases, Centers for Disease Control and Prevention, Atlanta, GA, USA; d Wilmer Eye Institute, Johns Hopkins University, Baltimore, MD, USA; e Muhimbili University, Dar es Salaam, Tanzania

**Keywords:** Antibody, chlamydia, mass drug administration, trachoma, trachomatis

## Abstract

***Purpose***: Trachoma, caused by repeated ocular infection with *Chlamydia trachomatis*, is the leading infectious cause of blindness worldwide and is targeted for elimination as a public health problem. We sought to determine whether a one-time azithromycin mass treatment would reduce trachomatous inflammation–follicular (TF) levels below the elimination threshold of 5% in communities with disease prevalence between 5 and 9.9%.

***Methods***: The study was conducted in 96 sub-village units (balozis) in the Kongwa district of Tanzania which were predicted from prior prevalence surveys to have TF between 5 and 9.9%. Balozis were randomly assigned to the intervention and control arms. The intervention arm received a single mass drug administration of azithromycin. At baseline and 12-month follow-up, ocular exams for trachoma, ocular swabs for detection of chlamydial DNA, and finger prick blood for analysis of anti-chlamydial antibody were taken.

***Results***: Comparison of baseline and 12-month follow-up showed no significant difference in the overall TF_1-9_ prevalence by balozi between control and treatment arms. In the treatment arm there was a significant reduction of ocular infection 12 months after treatment (*p* = 0.004) but no change in the control arm. No change in Pgp3-specific antibody responses were observed after treatment in the control or treatment arms. Anti-CT694 responses increased in both study arms (*p* = 0.009 for control arm and *p* = 0.04 for treatment arm).

***Conclusion***: These data suggest that a single round of MDA may not be sufficient to decrease TF levels below 5% when TF_1-9_ is between 5 and 9.9% at baseline.

## Introduction

Trachoma, caused by repeated ocular infection with *Chlamydia trachomatis (*CT), is the leading infectious cause of blindness worldwide and is responsible for visual impairment in 1.2 million people and 3% of blindness globally.^1–3^ Trachoma is endemic in 51 countries with 325 million people at risk of blinding disease. The burden of the disease falls disproportionately on poor communities and most cases of active trachoma—the presence of follicles and/or intense inflammation in the upper tarsal conjunctiva—occur among impoverished children in rural sub-Saharan Africa. Infection is transmitted from person to person through direct spread from eye to eye during close contact, direct or indirect spread of infected nasal or ocular secretion on fingers or clothes (fomites), and by eye-seeking flies.^4,5^ The World Health Organization (WHO) simplified grading scheme for trachoma recognizes distinct clinical signs of trachoma: trachomatous inflammation—follicular (TF); trachomatous inflammation—intense (TI); trachomatous scarring (TS); trachomatous trichiasis (TT); and corneal opacity (CO).^6^

To eliminate trachoma as a public health problem by 2020, the WHO-led Global Alliance for the Elimination of Trachoma by 2020 (GET 2020) advocates the implementation of the SAFE strategy, which encompasses surgery for trichiasis, mass distribution of antibiotics to treat infection, promotion of facial cleanliness to prevent disease transmission, and environmental improvement to increase access to water and sanitation.^7–9^ A key component of trachoma control programs is mass drug administration (MDA), comprised of annual treatment with oral azithromycin (AZT) for all individuals in any district (or evaluation unit) in which the prevalence of TF in 1–9-year-olds (TF_1-9_) is equal to or greater than 10%.^10^ The 2014 Preferred Practices for Zithromax® Mass Drug Administration recommends 7 years of MDA when TF_1-9_ is ≥50%, 5 years of MDA when TF_1-9_ is between 30 and 49.9%, 3 years of MDA when TF_1-9_ is between 10 and 29.9%, and implementation of the F and E component in all communities. The target threshold for trachoma elimination is TF_1-9_ of <5%. When TF_1-9_ is between 5 and 9.9% at baseline, either targeted treatment in affected subdistricts or MDA for a single year for the entire district is recommended, but historically these communities have fallen through the gaps of overburdened national programs. We sought to determine whether a one-time azithromycin MDA would be sufficient in communities with 5–9.9% TF_1-9_ to decrease TF_1-9_ levels below the elimination threshold of 5%. To accomplish this, we conducted a randomized controlled study of 96 balozis (neighborhood units) in the Kongwa District of Tanzania with anticipated TF_1-9_ prevalence of 5–9.9%, in which half of the balozis received a single dose of azithromycin, while the control arm did not. TF_1-9_, ocular infection, and anti-CT antibody responses were assessed at baseline and 12 months later.

## Methods

### Ethical considerations

This study adhered to the guidelines of the Declaration of Helsinki. Ethical approval for the study was granted by the Institutional Review Board (IRB) of the Institute for Medical Research (NIMR) Ethical Review Committee in Dar es Salaam, Tanzania and the Centers for Disease Control and Prevention, Atlanta, GA USA. Parental consent was obtained for all children aged 1–9 years enrolled in the study, and verbal assent was also obtained from children 7–9 years of age. No serious adverse events were associated with MDA.

### Study population and sample size calculation

The study was conducted in the Kongwa district in the Dodoma region of Tanzania in collaboration with the Kongwa Trachoma Project (KTP). Within Kongwa district, 96 balozis which had not been treated with azithromycin since 2009 and were predicted from prior prevalence surveys to have TF between 5 and 9.9% were selected for study. Balozis were randomly assigned to the intervention and control arms. The intervention arm received a single mass treatment with azithromycin given after baseline data collection and the control arm did not. The study end point was 12 months after baseline data collection. We expected to have on average 20 children aged 1–9 years per balozi, resulting in 960 children per study arm. At baseline and at the 12-month follow-up, a new census was conducted and a new random sample of children aged 1–9 were selected for participation in the study.

A sample of 48 balozis in each arm would achieve 83% power to detect a difference of 3.5% in the prevalence of TF_1-9_ between the treatment and control groups at the 0.05 significance level (alpha) using a two-sided t-test. These results are based on 2000 Monte Carlo samples from the null distributions: exponential with mean 6.5% in both arms, and the alternative distributions: exponential with mean 3% in the treatment arm and exponential with mean 6.5% in the control arm.

### Data collection

Baseline data collection occurred from October–December 2012 and the 12-month follow-up data collection occurred from October–December 2013. MDA was given between November 2012 and January 2013, approximately 1-2 weeks after field data collection from each village. A single oral dose of azithromycin was given at 20 mg/kg body weight, up to 1 g. Pregnant women and infants under the age of 6 months were instead offered tetracycline eye ointment for daily use for up to 6 weeks. Children aged 1–9 years who had parental consent for participation in the study were examined for clinical signs of trachoma by trained graders using 2.5 magnifying loupes, a torch (flashlight), and the simplified trachoma grading system.^6^ Ocular swabs were collected from the right eye for determination of ocular CT infection. Dried blood spots were prepared on TropBio filter paper (Cellbio, Australia) from finger prick blood to test for antibodies to CT antigens. The presence of ocular and/or nasal discharge and flies on the face was recorded by the examiner during trachoma evaluations.^11^ Parents or guardians of participants were asked about village health education messages related to facial cleanliness in the previous year.^11^ Ocular swabs were immediately placed in a cold chest and stored there until brought back to KTP at the end of each day, where they were stored at −20°C until shipped to CDC. Blood (approximately 60 μl) was collected onto filter paper and dried overnight, then packed into individual plastic bags and stored with dessicant at –20°C until shipped to CDC. All samples were stored at –20°C at CDC until analysis.

### Detection of anti-CT antibodies

The initial selection, expression, purification, and methodology for detection of IgG antibody against the CT-derived recombinant proteins CT694 and Pgp3 using multiplex bead array are described elsewhere.^12^ Briefly, serum was eluted from dried blood spots and then incubated with chemically-modified microspheres (Luminex Corp., Austin, TX, USA) conjugated to Pgp3 and CT694. After washing out unbound serum antibody, bound antibody was detected with biotinylated mouse anti-human IgG (clone H2; Southern Biotech, Birmingham, AL, USA) and biotinylated mouse anti-human IgG_4_ (clone HP6025; Invitrogen, South San Francisco, CA, USA), followed by *R*-phycoerythrin-labeled streptavidin (SAPE, Invitrogen, South San Francisco, CA, USA). Beads were suspended in 125 µl PBS, shaken, and PE emission was immediately read on a BioPlex 200 instrument (Bio-Rad, Hercules, CA, USA) equipped with Bio-Plex Manager 6.0 software (Bio-Rad). Specimens with coefficient of variation in duplicate wells greater than 15% were rerun. Cutoffs were determined as previously described.^12^

### Detection of ocular infection with CT

DNA was extracted using a modified High Pure PCR Template Preparation Kit (Roche Diagnostics Corporation, Indianapolis, IN, USA), following the manufacturer’s procedure. Briefly, the swabs were incubated in 640 μl extraction buffer for 1 hour at 72°C. After vigorous shaking, the eluate was transferred to the High Pure filter tubes provided in the kit and, following the washing steps, the DNA was eluted in 200 μl elution buffer. CT was detected by amplifying the *ompA* gene, which codes for the chlamydial major outer membrane protein, using a nested PCR protocol (Expand High Fidelity PCR System, Roche Diagnostics Corporation, Indianapolis, IN, USA) as previously described.^13^ Briefly, 10 μl of extracted DNA was used in the 1st PCR reaction, which contained primers CT90UF (5′-GGACATCTTGTCTGGCTTTAACT-3′) and CT220DR (5′-GCGCTCAAGTAGACCGATATAGTA-3′) located upstream and downstream, respectively, of the *omp1* coding region. The thermocycler profile was: 94°C, 30 seconds; 52°C, 1 minute; 72°C for 1 minute, for 40 cycles. Two μl of the 1st PCR reaction was used in a 2nd PCR reaction, which contained primers CT60UF (5′-GTCCCGCCAGAAAAAGATAG-3′) and CT80DR (CCAGAAACACGGATAGTGTTATTA-3′). The amplification products were separated on a 2.5% agarose gel. To confirm the specificity of the amplified product, the putative *ompA* fragments were purified using the QIAquick PCR Purification Kit (Qiagen Valencia, CA, USA) as instructed by the manufacturer, and sequenced with Big Dye Sequencing Terminator Kit and ABI Prism 377 automated sequencing system (PE-Applied Biosystems, Foster City, CA, USA), using the following sequencing primers: CT40F (5′-ATAGCGAGCACAAAGAGAGC-3′), CT419F (5′-TGGGATCGTTTTGATGTATT-3′) and CT902F (5′-TCCTTACATTGGAGTTAAATGGTC-3′).

### Statistical analysis

Data were entered using the LINKS system^14^ and downloaded to an MS Excel spreadsheet. Data were cleaned prior to analysis in STATA 13 (Stata Corporation, College Station, Texas). Data were aggregated by cluster (balozi) and prevalence of TF, ocular infection, and antibody responses calculated, with TF as the primary outcome. For demographic data, differences in outcomes at the individual level within each arm at baseline and 12-month follow-up were estimated using Pearson Chi-square except for the number of children per balozi, for which the Mann-Whitney test was used to compare medians. For trachoma assessments (i.e. TF, ocular infection, and antibody responses), the Mann-Whitney test was used to determine statistical differences of aggregate cluster data. The range and the interquartile range (IQR) were reported with the median. Statistical significance was determined at the 5% level.

## Results

### Study population characteristics

At baseline, 1621 children (48% male) were enrolled, and at the 12-month follow-up 1597 children (49% male) were enrolled (Table 1). The baseline group was comprised of 808 children in the treatment arm and 813 children in the control arm (Table 1). The median number of children per balozi was 16 for the control arm and 16.5 for the treatment arm (Table 1) at baseline. At the 12-month follow-up there were 812 children in the treatment arm and 785 children in the control arm (Table 1), and the median number of children per balozi was 15.5 for both arms (Table 1).

**Table 1.** Access to water and latrine usage among study participants, Kongwa District, Tanzania, October–December 2012 and 2013.

Characteristic	Baseline		*p*-value	12-month follow-up		*p*-value
	No treatment	Treatment		No treatment	Treatment	
No. of randomly sampled Balozi	48	48		48	48	
No. of children in the study	813	808		785	812	
Median (IQR) no. of children per Balozi	16 (10–25)	16.5 (10–24)	0.922	15.5 (10–23)	15.5 (11–24)	0.703
Distance from water source, *n* (%)						
<30 min	314 (39)	285 (35)	0.04	204 (26)	196 (24)	0.32
30 min–1 hour	263 (32)	310 (38)		433 (55)	478 (59)	
>1 hour	236 (29)	213 (26)		148 (19)	138 (17)	
Face-washing education, *n* (%)	813 (100)	808 (100)	–	330 (42)	395 (49)	0.008
Ocular/nasal discharge, *n* (%)	279 (34)	308 (38)	0.111	256 (32)	313 (39)	0.011
Flies on the face, *n* (%)	67 (8)	81 (10)	0.213	9 (1)	8 (1)	0.761
Pit latrine, *n* (%)	813 (100)	808 (100)	–	777 (99)	804 (99)	0.946

All participants reported face-washing health education in the previous year at baseline, but far fewer—42% in the control arm and 49% in the treatment arm—reported this at the 12-month follow-up (Table 1). On average, 36% of the children had ocular or nasal discharge at both baseline and 12-month follow-up with a statistically significant difference between the study arms at 12-month follow-up (Table 1). Average distance of household to a water source in the dry season was similar in the control and intervention groups at 12-month follow-up, although at baseline there was a slightly higher percent of participants with a 30 min–1 hour walk to a water source in the treatment group (Table 1). All participants reported access to a pit latrine in both arms of the study at baseline, and 99% reported access at the 12-month follow-up (Table 1).

### Effect of azithromycin MDA on trachoma assessments at 12-month follow-up

MDA coverage was 73%. For baseline data, median TF_1-9_ prevalence at the balozi level in the control arm was 6.0% and in the treatment arm was 4.3% (Table 2). Ocular CT infection had a median prevalence of 0% in both study arms at baseline (Table 2). Anti-Pgp3 prevalence was 16.7% in the control arm and 22.2% in the treatment arm, and anti-CT694 prevalence was 15.2% in the control arm and 19.6% in the treatment arm (Table 2).

**Table 2.** TF, C. trachomatis infection, and antibody responses at baseline and 12-month follow-up in clusters with and without mass drug administration.

	No treatment		*p*-value	Treatment		*p*-value
Balozi prevalence	Baseline	12-month follow-up		Baseline	12-month follow-up	
	Median (IQR)/(range)	Median (IQR)/(range)		Median (IQR)/(range)	Median (IQR)/(range)	
TF	6.0 (0–13.7)/(0–56.5)	8.2 (0–16.6)/(0–52.9)	0.479	4.3 (0–16.6)/(0–61.5)	9.3 (0–19.4)/(0–38.9)	0.263
*C. trachomatis* infection	0 (0–6.2)/(0–26.1)	0 (0–3.6)/(0–23.5)	0.370	0 (0–13.7)/(0–33.3)	0 (0–0)/(0–14.3)	0.004
anti-Pgp3 Ab	16.7 (7.7–29.9)/(0–70)	25.0 (8.4–40.0)/(0–88.2)	0.169	22.2 (10.3–40.0)/(0–84.6)	25.5 (15.1–51.8)/(0–84.2)	0.158
anti-CT694 Ab	15.2 (5.7–28.8)/0–70)	28.6 (13.0–44.0)/(0–91.3)	0.009	19.6 (11.2–37.2)/(0–84.6)	26.8 (14.6–54.2)/(0–81.8)	0.040

IQR, interquartile range; TF, trachomatous inflammation—follicular.

When comparing baseline and 12-month follow-up, there was no significant difference in the overall TF_1-9_ prevalence by balozi between the control and treatment arms (Table 2, *p* = 0.805). Ocular CT infection was also unchanged at the cluster level at the 12-month follow-up in the control arm, while in the treatment arm there was a significant reduction of ocular infection 12 months after treatment (*p* = 0.004; Table 2). No significant changes in Pgp3-specific antibody responses were observed before and after treatment in the control or treatment arms (Table 2). In contrast, anti-CT694 responses increased in both study arms (*p* = 0.009 for control arm and *p* = 0.04 for the treatment arm, Table 2). To determine if the single MDA had any effect on CT-specific Ab responses among one-year-olds, the age group most likely to be affected by a single MDA, we compared Pgp3 or CT694-specific responses in the treatment and control arms at the 12-month follow-up. Ab responses amongst one-year-olds showed slight, but not statistically significant, decreases for both Pgp3 (21.5% for control vs 10.9% for treatment arms, *p* = 0.651) and CT694 (20.0% for control vs 8.2% for treatment arms, *p* = 0.410).

## Discussion

The data presented here evaluated the effect of a single round of azithromycin MDA on TF, infection with CT, and antibody responses to CT antigens in balozis with an expected TF prevalence between 5 and 9.9%, which until recently (June 2015) was below the threshold for MDA as per WHO guidelines. In the current study, the prevalence of TF was low in both arms at baseline and there was no difference in the study arms following treatment; in fact in both arms there was a slight but non-statistically significant increase in TF at 12-month follow-up. While the median infection rate remained zero after MDA, there was a statistically significant decrease in the overall rates of ocular infection in balozis receiving MDA. A decrease in ocular Ct infection despite lingering TF following treatment has been previously observed^15–19^ although other studies have shown a decline of TF by 6 months after a single oral dose of azithromycin treatment.^20^ In the current study, TF rates increased slightly 12 months after treatment although there was a decrease in the range of infection prevalence among all balozis (the medians stayed at zero before and after treatment). TF rates were still below 10% TF in both study arms. These data suggest that a single round of MDA may not lower TF rates below 5% 1 year after treatment.

The percentage of children with detectable antibody responses to Pgp3 remained relatively constant at baseline and 12-month follow-up regardless of study arm, with a slight but not statistically significant increase at the 12-month follow-up. In contrast, responses to CT694 increased significantly in both study arms at the 12-month follow-up. While no change was observed in 1-year-olds, the age group, most likely to be affected by a single MDA, this may be due to small sample sizes (<10 per treatment arm) collected at baseline. Available data show that antibody responses to Pgp3 and CT694 are relatively long-lived^21^ and it is unlikely anti-CT antibody responses would decrease in response to MDA over such a short time frame. The increase in CT694 responses follows the general upward (but not statistically significant) trend of TF at the 12-month follow-up and so may simply be a result of new infections in the community.

Numerous factors may have influenced the inability of a single MDA to decrease TF levels below 5% in this study. Participants in the 12-month follow-up were less likely to report exposure to a face-washing educational campaign and were less likely to live within 30 minutes of a water source than those at baseline. MDA coverage overall was 73%, which may be lower than what is required for a single MDA to have a significant impact on TF as previously described.^22^ Alternatively, it may be that follicles, which can last for many months,^23^ are not themselves responsive to treatment and so may not disappear within the time frame under investigation.

At the WHO Trachoma Expert Committee (TEC) Meeting held in June 2015, it was decided that TEC would accept requests for azithromycin from countries to conduct one round of MDA in districts with TF_1-9_ between 5 and 9.9% at baseline. This decision gives programs a clearer path for managing evaluation units presenting with 5 and 9.9% TF_1-9_, which historically required additional targeted mapping, an approach that is programmatically less efficient and less acceptable to communities than MDA. The data presented here suggest that a single round of MDA may not be sufficient to drive TF levels below 5% when TF_1-9_ is between 5 and 9.9% at baseline; however, over the next few years more data will be collected to test the effectiveness of this strategy as programs implement single MDAs in low-prevalence settings.
